# A Relação entre a Relação Ácido Úrico/Albumina e a Espessura Média-Intimal da Carótida em Pacientes com Hipertensão

**DOI:** 10.36660/abc.20220819

**Published:** 2023-04-10

**Authors:** Faysal Şaylık, Tufan Çınar, Murat Selçuk, İbrahim Halil Tanboğa

**Affiliations:** 1 Departamento de Cardiologia Universidade de Ciências da Saúde Van Training and Education Hospital Van Turquia Departamento de Cardiologia, Universidade de Ciências da Saúde, Van Training and Education Hospital, Van – Turquia; 2 Departamento de Cardiologia Universidade de Ciências da Saúde Sultan II. Abdulhamid Han Training and Research Hospital Istanbul Turquia Departamento de Cardiologia, Universidade de Ciências da Saúde, Sultan II. Abdulhamid Han Training and Research Hospital, Istanbul – Turquia; 3 Departamento de Cardiologia e Bioestatística Istanbul Nisantasi University Istanbul Turquia Departamento de Cardiologia e Bioestatística, Istanbul Nisantasi University, Istanbul – Turquia

**Keywords:** Espessura Intima-Media Carotídea, Ácido úrico, Albuminas, hipertensão, Biomarcadores

## Abstract

**Fundamento:**

A hipertensão causa inflamação subendotelial e disfunção na aterosclerose resultante. A espessura média-intimal da carótida (EMIC) é um marcador útil de disfunção endotelial e aterosclerose. A razão ácido úrico/albumina (RUA) emergiu como um novo marcador para prever eventos cardiovasculares.

**Objetivo:**

Nosso objetivo foi investigar a associação da RUA com a EIMC em pacientes hipertensos.

**Método:**

Duzentos e dezesseis pacientes hipertensos consecutivos foram incluídos neste estudo prospectivo. Todos os pacientes foram submetidos a ultrassonografia de carótida para classificar baixos (EMIC < 0,9 mm) e altos (EMIC≥0,9 mm) grupos de EMIC. A capacidade preditiva da RUA para EMIC alta foi comparada com o índice de inflamação imune sistêmica (IIS), razão neutrófilo/linfócito (RNL), razão plaqueta/linfócito (RPL) e razão proteína C reativa/albumina (RCA). Um valor de p bilateral <0,05 foi aceito como estatisticamente significativo.

**Resultados:**

Os pacientes com EMIC alta eram mais velhos e tinham maior RUA, IIS, RNL e RCA do que baixo EMIC. Idade, RUA, IIS, RNL e RCA, mas não RPL, foram associados a EMIC alta. Na análise multivariada, idade, PCR, IIS e RUA foram preditores independentes de EMIC alta. A capacidade de discriminação de RUA foi maior do que ácido úrico, albumina, IIS, RNL e RCA, e RUA teve um ajuste de modelo maior do que essas variáveis. RUA teve maior melhoria aditiva na detecção de EMIC alta do que outras variáveis, conforme avaliado com melhoria de reclassificação líquida, MDI e estatísticas C. RUA também foi significativamente correlacionada com EMIC.

**Conclusão:**

RUA pode ser usado para prever EMIC alta e pode ser útil para estratificação de risco em pacientes hipertensos.

## Introdução

A prevalência de hipertensão (HA) é estimada em aproximadamente 30-45% em adultos em todo o mundo.^1^ Apesar da disponibilidade de opções de terapia avançada, a HA continua sendo um dos principais fatores de risco para morbidade e mortalidade cardiovascular.^1^ A aterosclerose é aceita como uma das principais causas de doenças cardiovasculares e cerebrovasculares.^2^ A HA leva à inflamação e disfunção subendotelial, que supostamente são a base patogenética da aterosclerose. É importante detectar aterosclerose subclínica de forma não invasiva em um estágio precoce para o prognóstico de pacientes hipertensos. A espessura média-intimal da carótida é um marcador objetivo de disfunção endotelial e aterosclerose.^3^ O valor preditivo da EIMC em doenças cardiovasculares foi avaliado em estudos anteriores.^4 , 5^

O ácido úrico sérico é um produto final do catabolismo das purinas. O alto nível sérico de ácido úrico tem um efeito pró-oxidante, é crítico no desenvolvimento da disfunção endotelial e leva a um risco cardiovascular elevado.^6^ Houve uma correlação significativa entre os níveis séricos de ácido úrico e EMIC.^7^ A albumina, um reagente negativo de fase aguda, tem um papel na manutenção da pressão plasmática oncótica e efeitos anti-inflamatórios. Níveis mais baixos de albumina sérica foram relacionados à aterosclerose carotídea e maior risco de mortalidade cardiovascular.^8 , 9^ Além disso, níveis baixos de albumina foram relatados como associados a EMIC alta.^10^

A relação ácido úrico/albumina sérica (RUA) foi recentemente relatada como um novo marcador associado a doenças cardiovasculares.^11^ Até onde sabemos, nenhuma prova implica que a RUA esteja relacionada à EIMC em pacientes hipertensos. Além disso, assumimos que a integração do ácido úrico sérico e da albumina em um único índice, RUA, predizia melhor a EIMC em pacientes hipertensos do que o ácido úrico sérico ou a albumina isoladamente ou marcadores inflamatórios bem conhecidos. Diante disso, este estudo teve como objetivo investigar a relação entre RUA e EIMC em pacientes hipertensos.

## Materiais e Métodos

### Coleção de dados

Duzentos e dezesseis pacientes diagnosticados com HA foram incluídos neste estudo transversal prospectivo. A definição de HA foi baseada na diretriz atual^12^ e foi diagnosticada como tendo pelo menos duas medidas de pressão arterial de consultório > 140/90 mmHg, ou uso de drogas anti-hipertensivas ou pressão arterial sistólica (PAS) média de 24 horas ≥ 130 mmHg e/ou pressão arterial diastólica média (PAD) ≥ 80 mmHg ou PAS diurna média ≥ 135 mmHg e/ou PAD ≥ 85 mmHg em uma monitoração ambulatorial bem-sucedida da pressão arterial. Pacientes com insuficiência cardíaca congestiva, hipertensão secundária, doença cardíaca valvular moderada a grave, doença arterial coronariana, doença renal ou hepática crônica, malignidade, infecção ativa, doença inflamatória crônica, aqueles que tomam medicamentos que afetam os níveis séricos de ácido úrico e/ou albumina e desnutrição foram excluídos do estudo.

Todas as características clínicas e demográficas dos pacientes foram anotadas durante a avaliação ambulatorial de rotina. O comitê de ética local aprovou o estudo (número aprovado:) e o consentimento informado por escrito foi obtido de todos os pacientes antes da inscrição. A pesquisa foi conduzida seguindo a Declaração de Helsinki, conforme revisada em 2008.

### Amostras de sangue

Amostras de sangue foram coletadas de todos os pacientes através da veia antecubital esquerda pela manhã, após um período de jejum noturno. Um analisador de hematologia Beckman Coulter LH 780 (Beckman Coulter, FL, EUA) foi usado para parâmetros hematológicos, e um Roche Cobas 6000 c501 (Roche, Mannheim, Alemanha) foi usado para parâmetros bioquímicos. A RUA foi calculada dividindo-se o nível sérico de ácido úrico pelo nível sérico de albumina. O índice de imuno-inflamação sistêmica (IIS) foi calculado com a seguinte fórmula; IIS = (plaqueta x neutrófilo) /linfócito. A razão neutrófilo/linfócito (RNL) foi calculada pela divisão de neutrófilo por linfócito, a razão plaqueta/linfócito (RPL) foi obtida pela divisão de plaqueta por linfócito, e a relação proteína C-reativa/albumina (RCA) foi obtida pela divisão da proteína C-reativa por albumina.

### Ultrassonografia modo B

Todos os pacientes foram examinados com um sistema de ultrassom de alta resolução (Toshiba Aplio 300 Toshiba Co. Ltd., Tóquio, Japão) para ambas as artérias carótidas comuns (CCA) direita e esquerda em posição supina por um ultrassonografista experiente que desconhecia os dados dos pacientes. O EMIC foi medido com um transdutor linear usando uma frequência de 10,0 MHz (8,0-12,0 MHz). As paredes anterior e posterior do CCA foram demonstradas longitudinalmente. De acordo com as diretrizes da American Society of Echocardiography Carotid Intima-Media Thickness Task Force, uma região proximal de um centímetro da bifurcação carotídea foi localizada e foram obtidas varreduras de alta resolução da parede oposta da artéria carótida comum bilateral (CCA).^13^ A EMIC foi obtida como a medida entre a borda de ataque da interface lúmen-íntima e a interface média-adventícia durante a diástole. A média EMIC foi calculada como a média das duas imagens de melhor qualidade de cada segmento CCA em ambos os lados. Um valor EMIC de0,9 foi considerado anormal.^1^

### Análise estatística

Todas as análises estatísticas foram feitas usando R-software v. 3.6.3 (software estatístico R, Instituto de Estatística e Matemática, Viena, Áustria). A distribuição normal foi verificada usando o Kolmogorov-Smirnov. As variáveis contínuas com distribuição normal foram relatadas como média e desvio padrão (DP) e com distribuição não normal como mediana (intervalo interquartil (IQR)). Os números e porcentagens foram usados para relatar os dados categóricos. O teste χ2 ou teste exato de Fisher foi usado para comparar variáveis categóricas entre os grupos, conforme apropriado. O teste t de amostra independente ou teste U de Mann-Whitney foi usado para comparar variáveis contínuas. A análise de regressão logística univariada foi realizada para detectar a associação das variáveis com o grupo de EMIC alta. Foi realizada análise de regressão logística multivariada com variáveis estatisticamente significativas na análise de regressão logística univariada. Para detectar a multicolinearidade, foram calculados os valores de VIF (fator de inflação de variância >3) e de tolerância (<0,1). Para avaliar a melhora na capacidade de discriminação de modelos para EMIC alta entre o modelo de linha de base com fatores de risco tradicionais (idade, sexo masculino, diabetes mellitus, tabagismo e hiperlipidemia) e o modelo aumentado com a adição de variáveis, incluindo RNL, RPL, RCA, IIS e RUA para o modelo de linha de base, foram calculados estatísticas de concordância de Harrell (c-statistics) com teste DeLong,^14^ melhoria de discriminação integrada (MDI) e melhoria de reclassificação líquida (MRL).^15^ A curva de características operacionais do receptor (ROC) foi utilizada para detectar a capacidade de discriminação das variáveis para detectar o grupo de EMIC alta. As comparações ROC foram feitas usando o teste de De-long. Uma análise de correlação de Spearman foi usada para detectar a associação de RUA sérico com EMIC. Calculamos o tamanho amostral mínimo necessário de um estudo anterior incorporando um tamanho de efeito de 0,75, probabilidade de erro alfa de 0,05 e um poder de 80%, resultando em 40 pacientes em cada braço.^16^ Os achados foram analisados usando um intervalo de confiança (IC) de 95% e um limite de significância de valor p < 0,05.

## Resultados

A Figura Central mostra os principais dados deste estudo. As características demográficas e os resultados laboratoriais dos grupos de alta (EMIC > 0,9 mm, n=75) e baixa (EMIC < 0,9 mm, n=141) foram apresentados na Tabela 1 . O grupo de alta EIMC era mais velho que o grupo de baixa EIMC. A contagem de neutrófilos, creatinina sérica, nível de ácido úrico, proteína C reativa (PCR), RUA, RCA, RNL, RPL e IIS foram maiores, e hemoglobina, albumina, relação albumina/creatinina foi menor no grupo EMIC alto do que no o grupo EMIC baixo.

**Figura Central:**
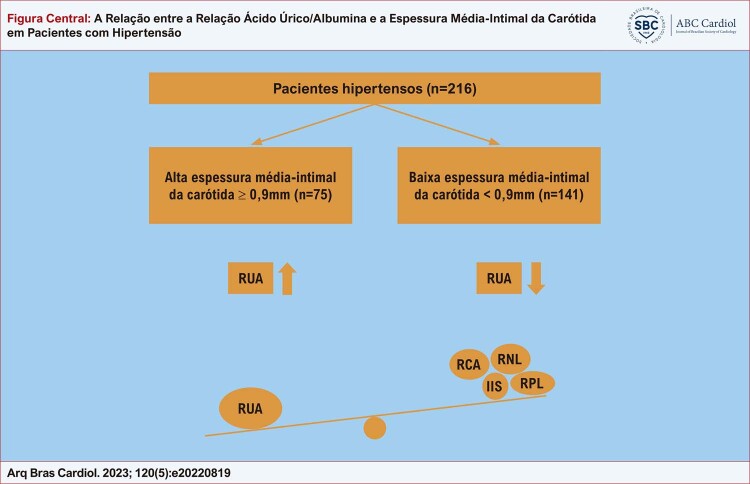
: A Relação entre a Relação Ácido Úrico/Albumina e a Espessura Média-Intimal da Carótida em Pacientes com Hipertensão

*Resumo dos principais dados deste estudo. RUA: relação ácido úrico/albumina; IIS: índice de imunoinflamação sistêmica; RNL: relação neutrófilo/linfócito; RPL: relação plaqueta/linfócito; RCA: relação proteína C reativa/albumina.*

**Tabela 1:** – Comparação de características demográficas e resultados laboratoriais entre grupos de alta e EMIC baixa

Variáveis	EMIC baixa	EMIC alta	valor-p
	(N=141)	(N=75)	
Idade, anos	59,0 (7,80)	62,6 (7,23)	0,001
Sexo masculino, n (%)	92 (65,2)	57 (76,0)	0,141
IMC, kg/m^2^	28,3 (3,04)	28,5 (2,82)	0,605
Diabetes mellitus, n (%)	50 (35,5)	33 (44,0)	0,280
Tabagismo, n (%)	44 (31,2)	30 (40,0)	0,252
Hiperlipidemia, n (%)	44 (31,2)	27 (36,0)	0,574
GB, x10^3^/µeu	9,01 (2,77)	9,39 (2,78)	0,334
Hemoglobina, g/dL	13,9 (2,70)	13.2 (2,21)	0,044
Plaquetas, x10^3^/µeu	279 (37,6)	289 (42,0)	0,093
Neutrófilo, x10^3^/µeu	4,80 (0,70)	5,30 (0,75)	<0,001
Linfócito, x10^3^/µeu	2,32 (0,70)	2,07 (0,64)	0,009
Creatinina, mg/dL	0,90 (0,04)	0,92 (0,06)	0,025
eGFR, kg/m^2^	98,7(8,8)	98,1 (6,7)	0,603
Colesterol total, mg/dL	201 (30,9)	205 (29.1)	0,296
LDL-colesterol, mg/dL	134 (37,3)	140 (26,1)	0,204
HDL-colesterol, mg/dL	40,5 (7,51)	38,7 (7,73)	0,107
Triglicerídeos, mg/dL	168 (11.3)	167 (14,7)	0,630
Ácido úrico, mg/dL	4,37 (3,83-4,99)	4,93 (4,56-5,48)	<0,001
Albumina, g/dL	3,82 (0,26)	3,70 (0,23)	0,001
Relação albumina/creatinina	4,23(0,35)	4,04(0,37)	<0,001
Proteína C reativa, mg/dL	3,22 (1,14)	3,75 (0,93)	<0,001
RUA	1,16 (0,25)	1,35 (0,19)	<0,001
RCA	0,87 (0,33)	1,11 (0,29)	<0,001
RNL	2,08 (1,65-2,73)	2,47 (1,96-3,32)	<0,001
RPL	119 (99,3-151)	143 (113-177)	0,002
IIS	573 (447-733)	835 (626-1055)	<0,001

*EMIC: espessura média-intimal da carótida; IMC: índice de massa corporal; GB: glóbulos brancos; eGFR: taxa de filtração glomerular estimada; LDL: baixa densidade; HDL: alta densidade; RUA: relação ácido úrico/albumina; RCA: C -relação proteína reativa/albumina; RNL: relação neutrófilo/linfócito; RPL: relação plaqueta/linfócito; IIS: índice de imunoinflamação sistêmica.*

A Tabela 2 compara características demográficas e resultados laboratoriais entre tercis RUA. O tercil RUA alto apresentou valores mais altos de neutrófilos, ácido úrico, RCA, RNL e IIS e albumina sérica e relação albumina/creatinina mais baixas do que o tercil RUA baixo.

**Tabela 2:** – Comparação de características demográficas e resultados laboratoriais entre os grupos RUA

	RUA < mediana	RUA ≥ mediana	valor-p
	(N=108)	(N=108)	
Idade, anos	59,3 (7,60)	61,1 (7,92)	0,093
Sexo masculino, n (%)	72 (66,7)	77 (71,3)	0,556
IMC, kg/m^2^	28,3 (2,87)	28,4 (3,06)	0,853
Diabetes mellitus, n (%)	39 (36,1)	44 (40,7)	0,576
Tabagismo, n (%)	34 (31,5)	40 (37,0)	0,473
Hiperlipidemia, n (%)	34 (31,5)	37 (34,3)	0,772
GB, x10^3^/µl	8,81 (2,88)	9,48 (2,63)	0,076
Hemoglobina, g/dL	13,7 (2,47)	13,6 (2,64)	0,796
Plaquetas, x10^3^/µl	278 (38,9)	287 (39,6)	0,112
Neutrófilo, x10^3^/µl	4,81 (0,72)	5,13 (0,76)	0,002
Linfócito, x10^3^/µl	2,30 (0,69)	2,17 (0,69)	0,175
Creatinina, mg/dL	0,91 (0,05)	0,91 (0,05)	0,277
eGFR, kg/m^2^	98,6 (8,4)	98,4 (7,7)	0,814
Colesterol total, mg/dL	203 (31,0)	202 (29,7)	0,808
LDL-colesterol, mg/dL	132 (34,3)	140 (33,1)	0,111
HDL-colesterol, mg/dL	40,1 (7,72)	39,6 (7,54)	0,638
Triglicerídeos, mg/dL	168 (11,9)	167 (13.2)	0,551
Ácido úrico, mg/dL	4,03 (3,65-4,43)	5,30 (4,85-5,64)	<0,001
Albumina, g/dL	3,86 (0,24)	3,69 (0,25)	<0,001
Relação albumina/creatinina	4,28(0,35)	4,05(0,36)	<0,001
Proteína C reativa, mg/dL	3,29 (1,18)	3,52 (0,99)	0,114
RUA	1,03 (0,15)	1,43 (0,14)	<0,001
RCA	0,88 (0,34)	1,03 (0,31)	0,001
RNL	2,14 (1,70-2,61)	2,37 (1,88-3,13)	0,027
RPL	121 (101-151)	128 (107-178)	0,086
IIS	595 (478-767)	720 (562-999)	0,002
EMIC, milímetros	0,73 (0,65-0,86)	0,89 (0,80-1,00)	<0,001

*EMIC: espessura média-intimal da carótida; IMC: índice de massa corporal; GB: glóbulos brancos; eGFR: taxa de filtração glomerular estimada; LDL: baixa densidade; HDL: alta densidade; RUA: relação ácido úrico/albumina; RCA: C -relação proteína reativa/albumina; RNL: relação neutrófilo/linfócito; RPL: relação plaqueta/linfócito; IIS: índice de imunoinflamação sistêmica.*

A análise de regressão logística univariada mostrou que idade, creatinina sérica, PCR, neutrófilos, linfócitos, IIS, RCA, RNL, RPL e RUA foram associados com EMIC alta. Na análise de regressão logística multivariada, idade, PCR, IIS e RUA foram preditores independentes de alta EIMC ( Tabela 3 ).

**Tabela 3:** – Análise de regressão logística para detecção do grupo EMIC alta

Variáveis	Regressão univariável			Regressão multivariável		
	
	OR	IC 95%	valor-p	OR	IC 95%	valor-p
Idade	1.066	1.026, 1.109	0,001	1.047	1.004, 1.094	0,034
Creatinina	1.074	1.015, 1.140	0,016	1.053	0,99, 1,124	0,111
Proteína C-reativa	1.591	1.212, 2.123	0,001	1.537	1.116, 2.155	0,010
Neutrófilo	2.670	1.757, 4.192	0,000	-	-	-
Linfócito	0,581	0,375, 0,883	0,012	-	-	-
IIS	1.002	1.001, 1.003	0,000	1.001	1.000, 1.002	0,019
RCA	11.47	4.346, 33.13	0,000	-	-	-
RNL	1.391	1.088, 1.845	0,016	-	-	-
RPL	1.003	1.000, 1.007	0,121	-	-	-
RUA	1.038	1.024, 1.054	0,000	1.032	1.016, 1.049	0,000

*EMIC: espessura média-intimal da carótida; RUA: relação ácido úrico/albumina; RCA: C -relação proteína reativa/albumina; RNL: relação neutrófilo/linfócito; RPL: relação plaqueta/linfócito; IIS: índice de imunoinflamação sistêmica; OR: Odds ratio; IC: intervalo de confiança.*

**Figura 1:**
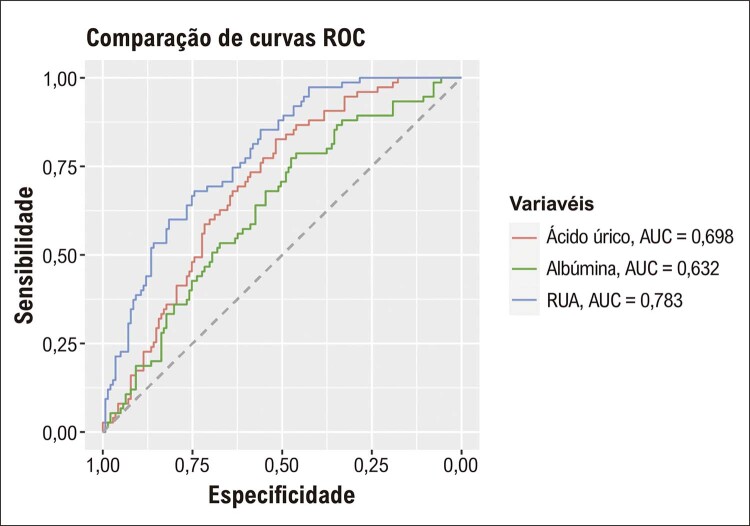
– Comparações ROC de ácido úrico sérico, albumina e RUA para detectar o grupo EMIC alta. ROC: características de operação do receptor; RUA: relação ácido úrico/albumina; EMIC: espessura média-intimal da carótida.

RUA teve maior χ2 do que todas as outras variáveis no modelo (χ2=14,8, p=0,0001), contribuindo com a maior capacidade preditiva do modelo completo na detecção de EMIC alta. As comparações do desempenho diagnóstico das variáveis para detectar EMIC alta foram apresentadas na Tabela 4 . RUA teve desempenho diagnóstico superior quando comparado a RPL, RNL, RCA e IIS. A análise ROC mostrou que a capacidade de discriminação de RUA para pacientes com alto EMIC de baixo EMIC foi maior do que outras variáveis, incluindo ácido úrico sérico, albumina, RNL, RPL, RCA e IIS ( Figuras 1 e 2 ). O gráfico de correlação entre RUA e EMIC mostrou uma correlação significativa, conforme demonstrado na Figura 3 .

**Tabela 4:** – Desempenhos diagnósticos de variáveis na detecção de EMIC alta

	AIC	BIC	-2LL	Nagelkarke R2	c estatística	Brier-Scaled
RPL	295	305	275,6	0,021	0,626	0,224
RNL	292	302	270,5	0,053	0,657	0,218
RCA	271	282	252.1	0,161	0,706	0,202
IIS	259	269	237	0,204	0,750	0,194
RUA	246	257	226	0,297	0,783	0,178

*AIC: critério de índice de Akaike; BIC: critério de índice bayesiano; LL: log-verossimilhança; RUA: relação ácido úrico/albumina; RCA: C -relação proteína reativa/albumina; RNL: relação neutrófilo/linfócito; RPL: relação plaqueta/linfócito; IIS: índice de imunoinflamação sistêmica.*

**Figura 2:**
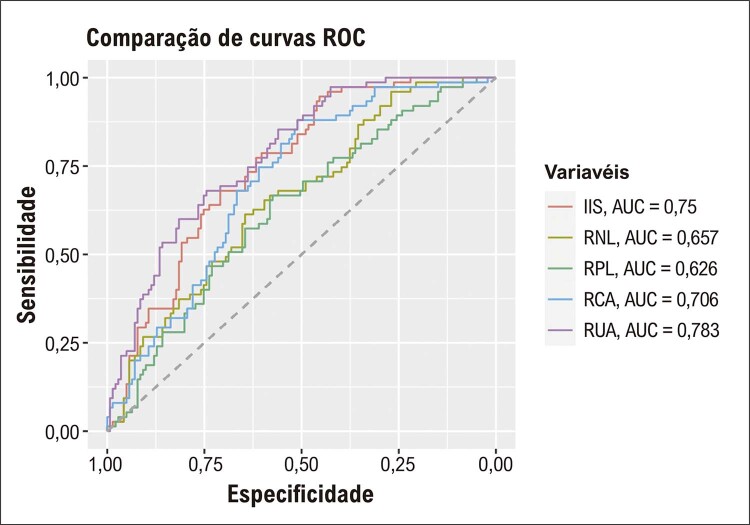
– Comparações ROC de RNL, RPL, RCA, IIS e RUA para detectar o grupo EMIC alta. ROC: características operacionais do receptor; RNL: razão neutrófilo/linfócito; RPL: razão plaqueta/linfócito; RCA: razão proteína c-reativa/albumina; IIS: índice de imunoinflamação sistêmica; RUA: razão ácido úrico/albumina; EMIC: espessura média-intimal da carótida.

**Figura 3:**
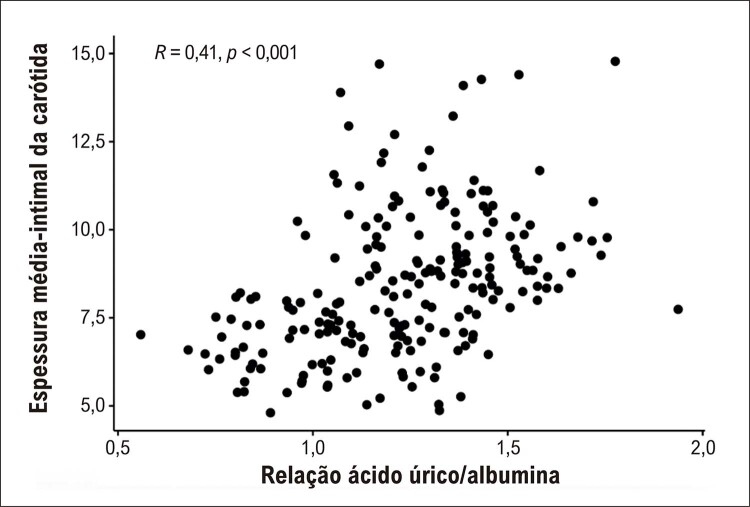
– O gráfico de correlação entre RUA e EMIC. RUA: relação ácido úrico/albumina. EMIC: espessura média-intimal da carótida.

### Valores preditivos adicionais após adicionar variáveis aos fatores de risco tradicionais para detectar EMIC alta

A adição de IIS a um modelo de linha de base com fatores de risco tradicionais (idade, sexo masculino, diabetes mellitus, tabagismo, hiperlipidemia) melhorou a detecção de EMIC alta, conforme demonstrado pelo aumento significativo na estatística-C ( Tabela 5 ). A reclassificação da adição de IIS aos fatores de risco tradicionais também mostrou uma melhoria de discriminação integrada (MDI) de 0,069 (p<0,001) com uma melhoria de 4,9% na melhoria de reclassificação líquida (MRL) (p=0,007). Adicionar RNL ao modelo de linha de base com fatores de risco tradicionais melhorou a detecção de EMIC alta com estatísticas c mais altas (0,698) e uma MDI de 0,025. No entanto, não houve benefício de RNL para reclassificação líquida (MRL =0,021, p-valor =0,079). A adição de RPL não melhorou a detecção de EMIC alta com fatores de risco tradicionais. A adição de RCA ao modelo de linha de base com fatores de risco tradicionais melhorou a detecção de EMIC alta com estatísticas c mais altas (0,753, valor p = 0,003) e uma MDI de 0,101 e melhoria de 10,1% na MRL (p = 0,003). Finalmente, adicionar RUA ao modelo de linha de base com fatores de risco tradicionais melhorou a detecção de EMIC alta com estatísticas c mais altas (0,765, valor p = 0,001) e uma MDI de 0,109 e a maior melhoria com 16,3% em MRL (p <0,001). Essas descobertas sugeriram que a adição de IIS, RCA e RUA pode detectar significativamente melhor a EMIC alta do que os fatores de risco tradicionais. Adicionar RUA ao modelo de linha de base com fatores de risco tradicionais melhorou a detecção de EMIC alta com estatísticas c mais altas (0,765, valor p = 0,001) e uma MDI de 0,109 e a maior melhoria com 16,3% em MRL (p <0,001). Essas descobertas sugeriram que a adição de IIS, RCA e RUA pode detectar significativamente melhor a EMIC alta do que os fatores de risco tradicionais.

**Tabela 5:** – Melhoria aditiva de variáveis para detectar EMIC alta adicionando aos fatores de risco tradicionais

	Diferença C-estatística	MRL (IC 95%)	IDI (IC 95%)
IIS	0,659-0,745 (p=0,002)	0,049 (0,014-0,086) (p=0,007)	0,069 0,036-0,102) (p<0,001)
RNL	0,659-0,698 (p= 0,038)	0,021 (-0,003-0,045) (p=0,079)	0,025 (0,003-0,047) (p=0,028)
RPL	0,659-0,671 (p=0,231)	0,007 (-0,007-0,021) (p= 0,316)	0,009 (-0,006-0,023) (p=0,231)
RCA	0,659-0,753 (p=0,003)	0,101 (0,035-0,167) (p=0,003)	0,101 (0,061-0,141) (p<0,001)
RUA	0,659-0,765 (p=0,001)	0,163 (0,102-0,224) (p<0,001)	0,109 (0,067-0,151) (p<0,001)

*MRL: melhoria de reclassificação líquida; IDI: índice de discriminação integrado; RUA: relação ácido úrico/albumina; RCA: C -relação proteína reativa/albumina; RNL: relação neutrófilo/linfócito; RPL: relação plaqueta/linfócito; IIS: índice de imunoinflamação sistêmica.*

## Discussão

Este estudo mostrou que os pacientes com alto EMIC tiveram maior RNL, RPL, RCA, IIS e RUA do que os pacientes com baixo EMIC. Todas essas variáveis foram associadas com EMIC alta. RUA teve um ajuste de modelo mais alto e capacidade discriminativa do que outras variáveis para EMIC alta. A melhoria aditiva para detectar EMIC alta adicionando RUA aos fatores de risco tradicionais foi maior com RUA do que com outras variáveis. Houve uma correlação significativa entre RUA e EMIC.

A EMIC, que pode ser facilmente obtida por ultrassonografia, tem sido utilizada como marcador prognóstico na doença aterosclerótica cardiovascular e pode predizer eventos clínicos futuros. Kawai et al. mostraram que a EIMC foi um preditor de AVC isquêmico e mortalidade em pacientes hipertensos.^17^ Zielinski et al. relataram que o grupo de EMIC alta teve um desfecho composto mais alto, incluindo morte, acidente vascular cerebral e infarto do miocárdio, quando comparado ao grupo de EMIC baixa.^18^ A inflamação desempenha um papel crítico no desenvolvimento de HA e aterosclerose.^8 , 19^ O ácido úrico sérico é um marcador de inflamação, e a relação entre ácido úrico sérico e doença cardiovascular já foi estudada antes. Níveis séricos mais elevados de ácido úrico foram relacionados à mortalidade cardiovascular ao longo de 10 anos de acompanhamento.^20^ Níveis séricos aumentados de ácido úrico foram encontrados em doenças metabólicas, incluindo obesidade, diabetes mellitus, hiperlipidemia e hipertensão.^21^ A hiperuricemia foi detectada em 25-47% dos pacientes hipertensos não tratados, podendo aumentar para 75% na HA maligna.^20^ Os mecanismos patogenéticos subjacentes da associação entre hiperuricemia e HA podem ser devidos ao sistema renina-angiotensina ativado, arteriopatia aferente renal e doença tubulointersticial.^22^ A hiperuricemia leva à disfunção endotelial, afetando a proliferação das células musculares lisas vasculares e inibindo a formação de óxido nítrico.^23^ A disfunção endotelial ocorre antes do desenvolvimento de complicações ateroscleróticas cardiovasculares.^24^ Dong et al. encontraram uma associação positiva entre os níveis séricos de ácido úrico e aterosclerose carotídea.^25^ Halcox et al. relataram uma associação significativa entre a disfunção endotelial e a progressão da EMIC ao longo de um período de acompanhamento de 6 anos.^24^ Em um estudo de Tavil et al., a EIMC foi detectada mais alta em hipertensos hiperuricêmicos do que em hipertensos normoisêmicos.^26^ Da mesma forma, o grupo RUA alto apresentou níveis de EMIC mais altos do que o grupo RUA baixo em nosso estudo.

Níveis baixos de albumina foram associados a um risco aumentado de eventos cardiovasculares.^8^ O valor prognóstico da albumina sérica em pacientes com síndrome coronariana aguda e insuficiência cardíaca foi relatado em estudos anteriores.^27 , 28^ Existem resultados contraditórios na literatura quanto à relação entre albumina sérica e aterosclerose carotídea. Yildirim et al. descobriram que pacientes com estenose grave da artéria carótida apresentavam níveis séricos de albumina mais baixos do que pacientes com estenose não grave.^8^ Em contraste, Folsom et al. não encontraram associação entre nível de albumina e EMIC.^29^ Em nosso estudo, EMIC alta apresentou níveis mais baixos de albumina, e a albumina foi associada ao grupo de EMIC alta.

Marcadores inflamatórios, como RCA, RNL, RPL e IIS, foram previamente estudados em pacientes com doença da artéria carótida. Yildirim et al. descobriram que o RCA era um preditor independente de alto EMIC.^8^ Cirakcíoglu et al. mostraram que o IIS estava relacionado de forma independente com EMIC alta.^30^ Mannarino et al. relataram que RNL foi correlacionada com EMIC, mas não com a progressão da EMIC ao longo dos anos.^31^ Assim como esse estudo, Lee et al. encontraram RNL como um preditor independente de EMIC alta.^32^ RPL também foi associada com EMIC em um estudo recente conduzido por Kaya et al.^33^ Após esses estudos, nosso estudo descobriu que RCA, RNL e IIS, mas não RPL, estavam associados a EMIC alta.

A hiperuricemia foi encontrada como relacionada ao maior estado inflamatório. Takir et al. mostraram que uma diminuição no nível sérico de ácido úrico estava associada à redução da inflamação.^34^ Zhou et al. descobriram que os níveis séricos de IL-6 e TNF-alfa eram maiores em pacientes hiperuricêmicos do que em controles, sugerindo papel adverso da inflamação em pacientes com hiperuricemia.^35^ Da mesma forma, os níveis de albumina sérica diminuem por uma inflamação ativada por uma elevação da PCR sérica, IL-6 e TNF-alfa. Assim, níveis mais altos de ácido úrico e níveis mais baixos de albumina, demonstrados com uma relação RUA mais alta, podem refletir um estado inflamatório mais elevado, uma causa subjacente de alta EIMC. RUA é um novo marcador recentemente relatado como um marcador preditivo de doença cardiovascular. Kalkan et al. relataram que a RUA foi um preditor independente de mortalidade em pacientes com infarto do miocárdio (IM) com elevação do segmento ST.^11^ Özgür et al. observaram que a RUA pode ser usada como um preditor independente de mortalidade em curto prazo em pacientes com lesão renal aguda.^36^ Çakmak et al. investigaram a RUA em pacientes com infarto do miocárdio sem supradesnivelamento do segmento ST e encontraram uma correlação entre a RUA e a extensão da doença arterial coronariana.^16^ Devido ao fato de um alto nível de ácido úrico e um baixo nível de albumina estarem relacionados à EIMC, buscamos investigar se a combinação desses marcadores prediz melhor a EIMC alta do que cada um isoladamente. Descobrimos que a RUA pode prever melhor a EMIC alta do que o ácido úrico e a albumina séricos e melhor do que todos os parâmetros inflamatórios mencionados acima, incluindo RNL, RPL, RCA e IIS.

Nosso estudo tem implicações clínicas proeminentes. RUA pode ser um marcador facilmente obtido e calculável para detectar pacientes hipertensos com EMIC alta melhor do que todos os outros parâmetros. Assim, os pacientes com alto risco para futuros eventos ateroscleróticos adversos podem ser detectados e observados de perto, e esses pacientes podem ser candidatos a opções de terapia mais intensivas.

### Limitações

O pequeno tamanho da amostra e um desenho de estudo de centro único foram as principais limitações deste estudo. Devido ao desenho do estudo transversal, houve uma falta de causalidade. Os pacientes não foram acompanhados em um desenho de estudo longitudinal. Portanto, não podemos relatar os eventos adversos da população do estudo, o impacto da RUA nesses desfechos e o impacto da RUA na progressão da EIMC ao longo do tempo. Finalmente, futuros estudos prospectivos, multicêntricos e longitudinais com amostras maiores são necessários para confirmar os resultados deste estudo.

## Conclusão

A RUA pode ser melhor do que seus componentes e outros marcadores inflamatórios como um preditor independente de EMIC alta em pacientes hipertensos.
