# Supplementary material for: A Relação entre a Relação Ácido Úrico/Albumina e a Espessura Média-Intimal da Carótida em Pacientes com Hipertensão
Source: Arq Bras Cardiol. 2023 Apr 10;120(5):e20220819. [Article in Portuguese] doi: 10.36660/abc.20220819 (PMC10124582; doi:10.36660/abc.20220819)
Supplement: Translation

# Translation: The Relationship between Uric Acid/Albumin Ratio and Carotid Intima-Media Thickness in Patients with Hypertension

**Authors:** Faysal Şaylık, Tufan Çınar, Murat Selçuk, İbrahim Halil Tanboğa

**Affiliations:** 1 Turkey Departamento de Cardiologia, Universidade de Ciências da Saúde, Van Training and Education Hospital, Van – Turkey; 2 Turkey Departamento de Cardiologia, Universidade de Ciências da Saúde, Sultan II. Abdulhamid Han Training and Research Hospital, Istanbul – Turkey; 3 Turkey Departamento de Cardiologia e Bioestatística, Istanbul Nisantasi University, Istanbul – Turkey

**Keywords:** Carotid Intima-Media Thickness, Uric Acid, Albumins, Hypertension, Biomarkers

## Abstract

**Background:**

Hypertension causes subendothelial inflammation and dysfunction in resulting atherosclerosis. Carotid intima-media thickness (CIMT) is a useful marker of endothelial dysfunction and atherosclerosis. The uric acid to albumin ratio (UAR) has emerged as a novel marker for predicting cardiovascular events.

**Objective:**

We aimed to investigate the association of UAR with CIMT in hypertensive patients.

**Methods:**

Two hundred sixteen consecutive hypertensive patients were enrolled in this prospective study. All patients underwent carotid ultrasonography to classify low (CIMT < 0.9 mm) and high (CIMT ≥ 0.9 mm) CIMT groups. The predictive ability of UAR for high CIMT was compared with systemic immune inflammation index (SII), neutrophil/lymphocyte ratio (NLR), platelet/lymphocyte ratio (PLR), and C-reactive protein/albumin ratio (CAR). A two-sided p-value <0.05 was accepted as statistically significant.

**Results:**

Patients with high CIMT were older and had higher UAR, SII, NLR, and CAR than low CIMT. Age, UAR, SII, NLR, and CAR, but not PLR, were associated with high CIMT. In multivariable analysis, age, CRP, SII, and UAR were independent predictors of high CIMT. The discrimination ability of UAR was higher than uric acid, albumin, SII, NLR, and CAR, and UAR had a higher model fit than those variables. UAR had higher additive improvement in detecting high CIMT than other variables, as assessed with net-reclassification improvement, IDI, and C-statistics. UAR was also significantly correlated with CIMT.

**Conclusion:**

UAR might be used to predict high CIMT and might be useful for risk stratification in hypertensive patients.

## Introduction

The prevalence of hypertension (HT) is estimated to be approximately 30-45% in adults worldwide.^1^ Despite the availability of advanced therapy choices, HT remains one of the main risk factors for cardiovascular morbidity and mortality.^1^ Atherosclerosis is accepted as a major cause of cardiovascular and cerebrovascular diseases.^2^ HT leads to subendothelial inflammation and dysfunction, which are supposed to be the pathogenetic basis of atherosclerosis. It is important to detect subclinical atherosclerosis non-invasively at an earlier stage for the prognosis of hypertensive patients. Carotid-intima media thickness is an objective marker of endothelial dysfunction and atherosclerosis.^3^ The predictive value of CIMT in cardiovascular diseases has been evaluated in previous studies.^4 , 5^

Serum uric acid is a final product of purine catabolism. High serum uric acid level has a pro-oxidant effect, is critical in developing endothelial dysfunction, and leads to an elevated cardiovascular risk.^6^ There was a significant correlation between serum uric acid levels and CIMT.^7^ Albumin, a negative acute phase reactant, has a role in maintaining oncotic plasma pressure and anti-inflammatory effects. Lower serum albumin levels were found to be related to carotid atherosclerosis and enhanced risk of cardiovascular mortality.^8 , 9^ Furthermore, low albumin levels were reported to be associated with high CIMT.^10^

Serum uric acid/albumin ratio (UAR) has been recently reported as a novel marker associated with cardiovascular diseases.^11^ To the best of our knowledge, no proof implicates that the UAR is related to CIMT in hypertensive patients. Furthermore, we assumed that integrating serum uric acid and albumin into a single index, UAR, would better predict CIMT in hypertensive patients than either serum uric acid or albumin alone or well-known inflammatory markers. As a result, this study aimed to investigate the relationship between UAR and CIMT in hypertensive patients.

## Materials and Methods

### Data collection

Two hundred sixteen patients diagnosed with HT were enrolled in this prospective cross-sectional study. The definition of HT was based on the current guideline^12^ and was diagnosed as having at least two office blood pressure measurements of >140/90 mmHg, or the use of anti-hypertensive drugs or mean 24-h systolic blood pressure (SBP) ≥ 130 mmHg and/or mean diastolic blood pressure (DBP) ≥ 80 mmHg or mean daytime SBP ≥ 135 mmHg and/or DBP ≥ 85 mmHg on a successful ambulatory blood pressure monitoring. Patients with congestive heart failure, secondary HT, moderate to severe valvular heart disease, coronary artery disease, chronic renal or liver disease, malignancy, active infection, chronic inflammatory disease, those taking drugs affecting serum uric acid and/or albumin levels, and malnutrition were excluded from the study.

All patients’ clinical and demographic characteristics were noted during the routine outpatient assessment. The local ethics committee approved the study (approved number:) and written informed consent was taken from all the patients before enrollment. The research was conducted following the Declaration of Helsinki as revised in 2008.

### Blood samples

Blood samples were taken from all patients via the left antecubital vein in the morning after an overnight fasting period. A Beckman Coulter LH 780 hematology analyzer (Beckman Coulter, FL, USA) was used for hematologic parameters, and a Roche Cobas 6000 c501 (Roche, Mannheim, Germany) was used for biochemical parameters. UAR was calculated by dividing serum uric acid level by serum albumin level. The systemic immune-inflammation index (SII) was calculated with the following formula; SII = (platelet x neutrophil)/lymphocyte. Neutrophil/lymphocyte ratio (NLR) was calculated by dividing neutrophil by lymphocyte, platelet/lymphocyte ratio (PLR) was obtained by dividing platelet by lymphocyte, and C-reactive protein/ albumin ratio (CAR) was obtained by dividing C-reactive protein by albumin.

### B mode ultrasonography

All patients were examined with a high-resolution ultrasound system (Toshiba Aplio 300 Toshiba Co. Ltd., Tokyo, Japan) for both right and left common carotid arteries (CCA) in a supine position by an experienced sonographer who was blinded to patients’ data. CIMT was measured with a linear transducer using a frequency of 10.0 MHz (8.0- 12.0 MHz). The anterior and posterior walls of CCA were demonstrated longitudinally. According to the American Society of Echocardiography Carotid Intima-Media Thickness Task Force guidelines, a one-centimeter proximal region of the carotid bifurcation was located, and high-resolution scans from the far wall of the bilateral common carotid artery (CCA) were obtained.^13^ CIMT was obtained as the measurement between the leading edge of the lumen-intima interface and the media-adventitia interface during diastole. The mean CIMT was calculated as the average of the two best-quality images of each CCA segment on both sides. A CIMT value of ≥ 0.9 was considered abnormal.^1^

### Statistical analysis

All statistical analyses were done using R-software v. 3.6.3 (R statistical software, Institute for Statistics and Mathematics, Vienna, Austria). The normal distribution was checked using the Kolmogorov–Smirnov. The continuous variables with normal distribution were reported as mean and standard deviation (SD), and with non-normal distribution as median (interquartile range (IQR)). The numbers and percentages were used to report the categorical data. The χ2 test or Fisher’s exact test was used to compare categorical variables between the groups, as appropriate. The independent sample t-test or Mann-Whitney U test was used to compare continuous variables. Univariable logistic regression analysis was performed to detect the association of variables with the high CIMT group. A multivariable logistic regression analysis was conducted with variables statistically significant in univariable logistic regression analysis. To detect multicollinearity, VIF (variance inflation factor >3) and tolerance (<0.1) values were calculated. To assess the improvement in discrimination ability of models for high CIMT between the baseline model with traditional risk factors (age, male gender, diabetes mellitus, cigarette smoking, and hyperlipidemia) and the augmented model with the addition of variables including, NLR, PLR, CAR, SII, and UAR to the baseline model, Harrell’s concordance statistics (c-statistics) with DeLong test,^14^ integrated discrimination improvement (IDI), and net reclassification improvement (NRI) were calculated.^15^ The receiver-operating characteristics (ROC) curve was used to detect the discrimination ability of variables for detecting the high CIMT group. The ROC comparisons were made using the De-long test. A Spearman correlation analysis was used to detect the association of serum UAR with CIMT. We calculated the minimum required sample size from a previous study by incorporating an effect size of 0.75, an alfa error probability of 0.05, and a power of 80 %, resulting in 40 patients in each arm.^16^ The findings were analyzed using a 95% confidence interval (CI) and a significance threshold of p-value < 0.05.

## Results

The central illustration shows the main data of this study. The demographic characteristics and laboratory results of high (CIMT > 0.9 mm, n=75) and low (CIMT < 0.9 mm, n=141) CIMT groups were presented in Table 1 . The high CIMT group was older than the low CIMT group. The neutrophil count, serum creatinine, uric acid level, C-reactive protein (CRP), UAR, CAR, NLR, PLR, and SII were higher, and hemoglobin, albumin, albumin/creatinine ratio was lower in the high CIMT group than in the low CIMT group.

**Central Illustration:** : The Relationship between Uric Acid/Albumin Ratio and Carotid Intima-Media Thickness in Patients with Hypertension

*The summary of the main data of this study. UAR: uric acid/albumin ratio; SII: systemic immune-inflammation index; NLR: neutrophil/lymphocyte ratio; PLR: platelet/lymphocyte ratio; CAR: C-reactive protein/albumin ratio.*

**Table 1:** – Comparisons of demographic features and laboratory results between high and low CIMT groups

Variables	Low CIMT	High CIMT	p-value
	(N=141)	(N=75)	
Age, years	59.0 (7.80)	62.6 (7.23)	0.001
Male gender, n (%)	92 (65.2)	57 (76.0)	0.141
BMI, kg/m^2^	28.3 (3.04)	28.5 (2.82)	0.605
Diabetes mellitus, n (%)	50 (35.5)	33 (44.0)	0.280
Smoking, n (%)	44 (31.2)	30 (40.0)	0.252
Hyperlipidemia, n (%)	44 (31.2)	27 (36.0)	0.574
WBC, x10^3^/µL	9.01 (2.77)	9.39 (2.78)	0.334
Hemoglobin, g/dL	13.9 (2.70)	13.2 (2.21)	0.044
Platelets, x10^3^/µL	279 (37.6)	289 (42.0)	0.093
Neutrophil, x10^3^/µL	4.80 (0.70)	5.30 (0.75)	<0.001
Lymphocyte, x10^3^/µL	2.32 (0.70)	2.07 (0.64)	0.009
Creatinine, mg/dL	0.90 (0.04)	0.92 (0.06)	0.025
eGFR, kg/m^2^	98.7(8.8)	98.1 (6.7)	0.603
Total cholesterol, mg/dL	201 (30.9)	205 (29.1)	0.296
LDL-cholesterol, mg/dL	134 (37.3)	140 (26.1)	0.204
HDL-cholesterol, mg/dL	40.5 (7.51)	38.7 (7.73)	0.107
Triglyceride, mg/dL	168 (11.3)	167 (14.7)	0.630
Uric acid, mg/dL	4.37 (3.83-4.99)	4.93 (4.56-5.48)	<0.001
Albumin, g/dL	3.82 (0.26)	3.70 (0.23)	0.001
Albumin/creatinine ratio	4.23(0.35)	4.04(0.37)	<0.001
C-reactive protein, mg/dL	3.22 (1.14)	3.75 (0.93)	<0.001
UAR	1.16 (0.25)	1.35 (0.19)	<0.001
CAR	0.87 (0.33)	1.11 (0.29)	<0.001
NLR	2.08 (1.65-2.73)	2.47 (1.96-3.32)	<0.001
PLR	119 (99.3-151)	143 (113-177)	0.002
SII	573 (447-733)	835 (626-1055)	<0.001

*CIMT: carotid-intima media thickness; BMI: body mass index; WBC: white blood cell; eGFR: estimated glomerular filtration rate; LDL: low-density; HDL: high-density; UAR: uric acid/albumin ratio; CAR: C-reactive protein/albumin ratio; NLR: neutrophil/lymphocyte ratio; PLR: platelet/lymphocyte ratio; SII: systemic immune-inflammation index.*

Table 2 compares demographic characteristics and laboratory results between UAR tertiles. High UAR tertile had higher neutrophil, uric acid, CAR, NLR, and SII values and lower serum albumin and albumin/creatinine ratio than low UAR tertile.

**Table 2:** – Comparisons of demographic features and laboratory results between UAR groups

	UAR < median	UAR ≥ median	p-value
	(N=108)	(N=108)	
Age, years	59.3 (7.60)	61.1 (7.92)	0.093
Male gender, n (%)	72 (66.7)	77 (71.3)	0.556
BMI, kg/m^2^	28.3 (2.87)	28.4 (3.06)	0.853
Diabetes mellitus, n (%)	39 (36.1)	44 (40.7)	0.576
Smoking, n (%)	34 (31.5)	40 (37.0)	0.473
Hyperlipidemia, n (%)	34 (31.5)	37 (34.3)	0.772
WBC, x10^3^/µl	8.81 (2.88)	9.48 (2.63)	0.076
Hemoglobin, g/dL	13.7 (2.47)	13.6 (2.64)	0.796
Platelets, x10^3^/µl	278 (38.9)	287 (39.6)	0.112
Neutrophil, x10^3^/µl	4.81 (0.72)	5.13 (0.76)	0.002
Lymphocyte, x10^3^/µl	2.30 (0.69)	2.17 (0.69)	0.175
Creatinine, mg/dL	0.91 (0.05)	0.91 (0.05)	0.277
eGFR, kg/m^2^	98.6 (8.4)	98.4 (7.7)	0.814
Total cholesterol, mg/dL	203 (31.0)	202 (29.7)	0.808
LDL-cholesterol, mg/dL	132 (34.3)	140 (33.1)	0.111
HDL-cholesterol, mg/dL	40.1 (7.72)	39.6 (7.54)	0.638
Triglyceride, mg/dL	168 (11.9)	167 (13.2)	0.551
Uric acid, mg/dL	4.03 (3.65-4.43)	5.30 (4.85-5.64)	<0.001
Albumin, g/dL	3.86 (0.24)	3.69 (0.25)	<0.001
Albumin/creatinine ratio	4.28(0.35)	4.05(0.36)	<0.001
C-reactive protein, mg/dL	3.29 (1.18)	3.52 (0.99)	0.114
UAR	1.03 (0.15)	1.43 (0.14)	<0.001
CAR	0.88 (0.34)	1.03 (0.31)	0.001
NLR	2.14 (1.70-2.61)	2.37 (1.88-3.13)	0.027
PLR	121 (101-151)	128 (107-178)	0.086
SII	595 (478-767)	720 (562-999)	0.002
CIMT, mm	0.73 (0.65-0.86)	0.89 (0.80-1.00)	<0.001

*CIMT: carotid-intima media thickness; BMI: body mass index; WBC: white blood cell; eGFR: estimated glomerular filtration rate; LDL: low-density; HDL: high-density; UAR: uric acid/albumin ratio; CAR: C-reactive protein/albumin ratio; NLR: neutrophil/lymphocyte ratio; PLR: platelet/lymphocyte ratio; SII: systemic immune-inflammation index.*

Univariable logistic regression analysis showed that age, serum creatinine, CRP, neutrophil, lymphocyte, SII, CAR, NLR, PLR, and UAR was associated with high CIMT. In multivariable logistic regression analysis, age, CRP, SII, and UAR were independent predictors of high CIMT ( Table 3 ).

**Table 3:** – Logistic regression analysis for detecting high CIMT group

Variables	Univariable regression			Multivariable regression		
	
	OR	95% CI	p-value	OR	95% CI	p-value
Age	1.066	1.026, 1.109	0,001	1.047	1.004, 1.094	0,034
Creatinine	1.074	1.015, 1.140	0,016	1.053	0,99, 1,124	0,111
C-reactive protein	1.591	1.212, 2.123	0,001	1.537	1.116, 2.155	0,010
Neutrophil	2.670	1.757, 4.192	0,000	-	-	-
Lymphocyte	0,581	0,375, 0,883	0,012	-	-	-
SII	1.002	1.001, 1.003	0,000	1.001	1.000, 1.002	0,019
CAR	11.47	4.346, 33.13	0,000	-	-	-
NLR	1.391	1.088, 1.845	0,016	-	-	-
PLR	1.003	1.000, 1.007	0,121	-	-	-
UAR	1.038	1.024, 1.054	0,000	1.032	1.016, 1.049	0,000

*OR: odds ratio; CI: confidence interval; UAR: uric acid/albumin ratio; CAR: C-reactive protein/albumin ratio; NLR: neutrophil/lymphocyte ratio; PLR: platelet/lymphocyte ratio; SII: systemic immune-inflammation index.*

UAR had a higher χ2 than all other variables in the model (χ2=14.8, p=0.0001) by contributing the highest predictive ability of the full model in detecting high CIMT. Diagnostic performance comparisons of variables for detecting high CIMT were presented in Table 4 . UAR had higher diagnostic performance when compared to PLR, NLR, CAR, and SII. ROC analysis showed that the discrimination ability of UAR for patients with high CIMT from low CIMT was higher than other variables, including serum uric acid, albumin, NLR, PLR, CAR, and SII ( Figures 1 and 2 ). The correlation graph between UAR and CIMT showed a significant correlation, as demonstrated in Figure 3 .

**Table 4:** – Diagnostic performances of variables in detecting high CIMT

	AIC	BIC	-2LL	Nagelkarke R2	c statistic	Brier-Scaled
PLR	295	305	275.6	0.021	0.626	0.224
NLR	292	302	270.5	0.053	0.657	0.218
CAR	271	282	252.1	0.161	0.706	0.202
SII	259	269	237	0.204	0.750	0.194
UAR	246	257	226	0.297	0.783	0.178

*AIC: Akaike-index criterion; BIC: bayesian index criterion; LL: log-likelihood; UAR: uric acid/albumin ratio; CAR: C-reactive protein/albumin ratio; NLR: neutrophil/lymphocyte ratio; PLR: platelet/lymphocyte ratio; SII: systemic immune-inflammation index.*

**Figure 1:** – The ROC comparisons of serum uric acid, albumin, and UAR for detecting the high CIMT group. ROC: receiver-operating characteristics; UAR: uric acid/albumin ratio; CIMT: carotid-intima media thickness.

**Figure 2:** – The ROC comparisons of NLR, PLR, CAR, SII, and UAR for detecting the high CIMT group. ROC: receiver-operating characteristics; NLR: neutrophil/lymphocyte ratio; PLR: platelet/lymphocyte ratio; CAR: c-reactive protein/albumin ratio; SII: systemic immune-inflammation index; UAR: uric acid/albumin ratio; CIMT: carotid-intima media thickness.

**Figure 3:** – The correlation graph between UAR and CIMT. UAR: uric acid/albumin ratio. CIMT: carotid-intima media thickness.

### Additional predictive values after adding variables to the traditional risk factors for detecting high CIMT

Adding SII to a baseline model with traditional risk factors (age, male gender, diabetes mellitus, cigarette smoking, hyperlipidemia) improved the detection of high CIMT, as demonstrated by the significant increase in the C-statistics ( Table 5 ). Reclassification of adding SII to the traditional risk factors also showed an integrated discrimination improvement (IDI) of 0.069 (p<0.001) with a 4.9 % improvement in net reclassification improvement (NRI) (p=0.007). Adding NLR to the baseline model with traditional risk factors improved the detection of high CIMT with higher c-statistics (0.698) and an IDI of 0.025. However, there was no benefit of NLR for net reclassification (NRI =0.021, p=0.079). Adding PLR did not improve the detection of high CIMT with traditional risk factors. Adding CAR to the baseline model with traditional risk factors improved the detection of high CIMT with higher c-statistics (0.753, p=0.003) and an IDI of 0.101 and 10.1 % improvement in NRI (p=0.003). Finally, adding UAR to the baseline model with traditional risk factors improved the detection of high CIMT with higher c-statistics (0.765, p=0.001) and an IDI of 0.109 and the highest improvement with 16.3 % in NRI (p<0.001). These findings suggested that adding SII, CAR, and UAR might significantly better detect high CIMT than traditional risk factors.

**Table 5:** – Additive improvement of variables for detecting high CIMT by adding to the traditional risk factors

	C-statistic difference	NRI (95 % CI)	IDI (95 % CI)
SII	0.659-0.745 (p=0.002)	0.049 (0.014-0.086) (p=0.007)	0.069 0.036-0.102) (p<0.001)
NLR	0.659-0.698 (p= 0.038)	0.021 (-0.003-0.045) (p=0.079)	0.025 (0.003-0.047) (p=0.028)
PLR	0.659-0.671 (p=0.231)	0.007 (-0.007-0.021) (p= 0.316)	0.009 (-0.006-0.023) (p=0.231)
CAR	0.659-0.753 (p=0.003)	0.101 (0.035-0.167) (p=0.003)	0.101 (0.061-0.141) (p<0.001)
UAR	0.659-0.765 (p=0.001)	0.163 (0.102-0.224) (p<0.001)	0.109 (0.067-0.151) (p<0.001)

*NRI: net-reclassification improvement; IDI: integrated discrimination index; UAR: uric acid/albumin ratio; CAR: C-reactive protein/albumin ratio; NLR: neutrophil/lymphocyte ratio; PLR: platelet/lymphocyte ratio; SII: systemic immune-inflammation index.*

## Discussion

This study showed that patients with high CIMT had higher NLR, PLR, CAR, SII, and UAR than low CIMT patients. All those variables were associated with high CIMT. UAR had a higher model fit and discriminative ability than other variables for high CIMT. The additive improvement for detecting high CIMT by adding UAR to the traditional risk factors was higher with UAR than with other variables. There was a significant correlation between UAR and CIMT.

CIMT, which can be easily obtained by ultrasonography, has been used as a prognostic marker in cardiovascular atherosclerotic disease and can predict future clinical events. Kawai et al. showed that CIMT was a predictor of ischemic stroke and mortality in hypertensive patients.^17^ Zielinski et al. reported that the high CIMT group had a higher composite end-point, including death, stroke, and myocardial infarction when compared to the low CIMT group.^18^ Inflammation plays a critical role in developing HT and atherosclerosis.^8 , 19^ Serum uric acid is a marker of inflammation, and the relationship between serum uric acid and cardiovascular disease has been studied before. Higher serum uric acid levels were found to be related to cardiovascular mortality over 10 years of follow-up.^20^ Increased serum uric acid levels were found in metabolic diseases, including obesity, diabetes mellitus, hyperlipidemia, and HT.^21^ Hyperuricemia was detected in 25-47 % of untreated hypertensive patients, and that can rise to 75 % in malignant HT.^20^ The underlying pathogenetic mechanisms of the association between hyperuricemia and HT might be due to the activated renin-angiotensin system, renal afferent arteriopathy, and tubulointerstitial disease.^22^ Hyperuricemia leads to endothelial dysfunction by affecting vascular smooth muscle cell proliferation and inhibiting nitric oxide formation.^23^ Endothelial dysfunction occurs earlier before the development of cardiovascular atherosclerotic complications.^24^ Dong et al. found a positive association between serum uric acid levels and carotid atherosclerosis.^25^ Halcox et al. reported a significant association between endothelial dysfunction and the progression of CIMT over a 6-year follow-up period.^24^ In a study by Tavil et al., the CIMT was detected higher in hyperuricemic hypertensive patients than in normourisemic hypertensive patients.^26^ Similarly, the high UAR group had higher CIMT levels than the low UAR group in our study.

Low albumin levels were associated with an increased risk of cardiovascular events.^8^ The prognostic value of serum albumin in patients with acute coronary syndrome and heart failure was reported in previous studies.^27 , 28^ There were contradictory results regarding the relationship between serum albumin and carotid atherosclerosis in the literature. Yildirim et al. found that patients with severe carotid artery stenosis had lower serum albumin levels than patients with non-severe stenosis.^8^ In contrast, Folsom et al. found no association between albumin level and CIMT.^29^ In our study, high CIMT had lower albumin levels, and albumin was associated with the high CIMT group.

Inflammatory markers, such as CAR, NLR, PLR, and SII, have been previously studied in patients with carotid artery disease. Yildirim et al. found that CAR was an independent predictor of high CIMT.^8^ Cirakcıoglu et al. showed that SII was independently related to high CIMT.^30^ Mannarino et al. reported that NLR was correlated with CIMT but not with CIMT progression over the years.^31^ Like that study, Lee et al. found NLR as an independent predictor of high CIMT.^32^ PLR was also associated with CIMT in a recent study conducted by Kaya et al.^33^ Following those studies, our study found that CAR, NLR, and SII but not PLR were associated with high CIMT.

Hyperuricemia was found as related to higher inflammatory status. Takir et al. showed that a decrease in serum uric acid level was associated with reduced inflammation.^34^ Zhou et al. found that serum IL-6 and TNF-alfa levels were higher in hyperuricemic patients than in controls, suggesting inflammation’s adverse role in patients with hyperuricemia.^35^ Similarly, serum albumin levels decrease by an activated inflammation by an elevation in serum CRP, IL-6, and TNF-alfa. So, higher levels of uric acid and lower levels of albumin, demonstrated with a higher UAR ratio, could reflect a higher inflammatory status, an underlying cause of high CIMT. UAR is a novel marker recently reported as a predictive marker of cardiovascular disease. Kalkan et al. reported that UAR was an independent predictor of mortality in patients with ST-segment elevated myocardial infarction (MI).^11^ Ozgur et al. noted that UAR might be used as an independent predictor of short-term mortality in acute kidney injury patients.^36^ Cakmak et al. investigated the UAR in non-ST segment elevation MI patients and found a correlation between UAR and the extent of coronary artery disease.^16^ Due to a high level of uric acid and a low level of albumin being related to CIMT, we aimed to investigate whether the combination of these markers predicts high CIMT better than each alone. We found that UAR might better predict high CIMT than serum uric acid and albumin and better than all the above-mentioned inflammatory parameters, including NLR, PLR, CAR, and SII.

Our study has prominent clinical implications. UAR might be an easily obtained and calculable marker for detecting hypertensive patients with high CIMT better than all other parameters. So, the patients with a high risk for future adverse atherosclerotic events might be detected and observed closely, and these patients might be candidates for more intensive therapy options.

### Limitations

The small sample size and a single-center study design were the major limitations of this study. Due to the cross-sectional study design, there was a lack of causality. The patients were not followed-up in a longitudinal study design. Thus, we cannot report the study population’s adverse events, UAR’s impact on these outcomes, and the impact of UAR on CIMT progression over time. Finally, future prospective, multicenter, and longitudinal studies with larger samples are needed to confirm the results of this study.

## Conclusion

UAR might be better than its components and other inflammatory markers as an independent predictor of high CIMT in hypertensive patients.
